# P124: Increase in alcohol-based hand rub consumption over a period of five years in German hospitals

**DOI:** 10.1186/2047-2994-2-S1-P124

**Published:** 2013-06-20

**Authors:** M Behnke, C Reichardt, P Gastmeier

**Affiliations:** 1 Institute of Hygiene and Environmental Medicine, Charité, Berlin, Germany

## Introduction

In 2008, the German national nosocomial infection surveillance system (KISS) introduced a new surveillance module: HAND-KISS. HAND-KISS is a unit-based system for the surveillance of alcohol-based hand rub consumption (AHC). On the basis of HAND-KISS data, we studied the change in AHC between 2008 and 2012.

## Methods

Participating hospitals annually transfer data on patient days and AHC per unit to the surveillance system. HAND-KISS then provides the data as AHC in milliliter (ml) per patient day (PD) stratified by unit type (intensive care unit and normal ward) and unit speciality (medical, surgical, pediatrics, neonatal etc.). The data on all participating hospitals and units are summarized and published anonymously as HAND-KISS reference data on a yearly basis. To assess AHC changes over the years, we selected all hospitals which continuously provided surveillance data over a period of five years (2008 to 2012). Within this cohort we selected units which continuously provided data to HAND-KISS. These units were further stratified in intensive care units (ICU) and normal wards. For all groups we estimated the median AHC (interquartile range, IQR) for every year and compared the results.

## Results

Five hundred and four hospitals including 4,762 units provided AHC data for 2008, and 791 hospitals with 9,256 units transmitted AHC data for 2012. One hundred seventy-seven hospitals, 140 ICUs and 165 normal wards within these hospitals continuously provided surveillance data over a 5 year period. In 2008, the median AHC in the ICUs was 72 ml/PD (IQR, 58 ml/PD - 93 ml/PD), and in 2012 the result was 97 ml/PD (IQR, 77 ml/PD - 124 ml/PD). The median AHC was 18 ml/PD (IQR, 15 ml/PD - 25 ml/PD) in 2008 and 27 ml/PD (IQR, 23 ml/PD - 36 ml/PD) in 2012. In 2008 the median AHC on normal wards was 16 ml/PD (IQR, 13 ml/PD - 18 ml/PD) and in 2012 the median AHC was 24 ml/PD (IQR 19 ml/PD - 28 ml/PD). There was an increase of 46% in AHC within the group of analyzed hospitals. On the analyzed ICUs AHC increased by 36% and on normal wards by 48%.

## Conclusion

HAND-KISS observed an increase in AHC and therewith increased attention to hand hygiene in the participating hospitals. HAND-KISS as unit based surveillance system for AHC provides a benchmarking tool to characterize hand hygiene behavior in an individual institution.

## Disclosure of interest

None declared

